# Exosome-Like Nanoparticles from Indonesian Red and Emprit Ginger Varieties Suppress LPS-Induced IL-6 Production in RAW 264.7 Macrophages

**DOI:** 10.34172/apb.42675

**Published:** 2025-03-23

**Authors:** Daisy Ramadhani Muhammad, Natasya Emmanuela, Iriawati Iriawati, Christofora Hanny Wijaya, Ika Dewi Ana, Triarti Dewi Kencana Wungu, Diah Ratnadewi, Hiroshi Takemori, Anggraini Barlian

**Affiliations:** ^1^Department of Biotechnology, School of Life Sciences and Technology, Institut Teknologi Bandung, Bandung, West Java, Indonesia; ^2^Department of Food Science and Technology, Faculty of Agricultural Engineering and Technology, IPB University, Bogor, Indonesia; ^3^Department of Dental Biomedical Sciences, Faculty of Dentistry, Universitas Gadjah Mada, Yogyakarta, Indonesia; ^4^Department of Physics, Faculty of Mathematics and Natural Sciences, Institut Teknologi Bandung, Bandung, Indonesia; ^5^Research Center for Nanoscience and Nanotechnology, Institut Teknologi Bandung, West Java, Indonesia; ^6^Department of Biology, Faculty of Mathematics and Natural Sciences, IPB University, Bogor, Indonesia; ^7^Department of Chemistry and Biomolecular Science, Faculty of Engineering, Gifu University, Gifu, Japan; ^8^Scientific Imaging Center (SIC) ITB, Institut Teknologi Bandung, Bandung, Indonesia

**Keywords:** Exosome, Interleukin 6, Anti-inflammatory, Macrophage, *Zingiber officinale*

## Abstract

**Purpose:**

Studies have shown the potential of exosomes as therapeutic agents with anti-inflammatory properties. However, the clinical application of mammalian-derived exosomes is hindered by mass production challenges and strict regulations. Plant-derived exosome-like nanoparticles (PELNs) are a more economical alternative possessing a similar therapeutic potential. Ginger is a readily available plant with components that are clinically proven to inhibit inflammation. Therefore, it is interesting to investigate the potential of red ginger and emprit ginger, cultivated varieties in Indonesia possessing the most potent anti-inflammatory activities, as a PELN source for anti-inflammatory therapy.

**Methods:**

In this work, PELNs from the rhizomes of red ginger (RG-ELN) and emprit ginger (EG-ELN) were obtained through differential centrifugation and polymer precipitation using PEG6000. The PELNs were characterized by transmission electron microscopy (TEM), dynamic light scattering (DLS), and bicinchoninic acid assay. Their internalization and effect on RAW 246.7 cell viability were also assessed. The anti-inflammatory potential of PELNs was investigated by assessing interleukin 6 (IL-6) expression of lipopolysaccharide (LPS)-stimulated macrophages treated with RG-ELN and EG-ELN.

**Results:**

Both RG-ELN and EG-ELN exhibited cup-shaped morphologies with average sizes of 195.83±1.35 and 194.40±8.40 nm, respectively. Both PELNs can be internalized within 2 h and did not significantly affect RAW 264.7 cell viability after 24 h. The reverse transcription quantitative real-time polymerase chain reaction and enzyme-linked immunosorbent assay results indicated a significantly lower expression and secretion of IL-6 in the macrophage cells pre-treated with RG-ELN and EG-ELN.

**Conclusion:**

The RG-ELN and EG-ELN samples were successfully obtained through the polymer precipitation method, as confirmed by the TEM and DLS results which aligned with typical PELN characteristics. The pre-treatment of RG-ELN and EG-ELN to activated RAW 264.7 cells decreased the pro-inflammatory cytokine IL-6 expression relative to activated controls.

## Introduction

 Inflammation is a vital protective response that might result in various health complications when dysregulated. Within the last two decades, epidemiological studies have revealed a significant increase in inflammatory diseases, with their prevalence expected to continuously rise for the next 30 years.^1^ However, conventional approaches in managing inflammatory diseases involve the usage of anti-inflammatory and immunosuppressive drugs, which possesses several drawbacks such as toxicity and drug resistance,^2^ as well as an increased infection risk caused by the prolonged suppression of the immune system.^3^ Therefore, research regarding alternative treatment methods that are capable of influencing the immune system without causing side effects associated with conventional drug use is urgently needed.

 Exosomes are a type of extracellular vesicle ranging in size between 30 and 150 nm. They are secreted by cells through the endosomal pathways as a form of intercellular communication.^4^ Exosomes derived from certain cells, such as mesenchymal stem cells (MSCs), may mimic the immunomodulatory properties of their source cells; hence, they are now widely studied for use in cell-free therapies, including those aiming to treat inflammatory diseases. The therapeutic effect of MSC-derived exosomes is mainly mediated by their cargo, namely, bioactive molecules, such as metabolites, lipids, proteins, and various kinds of RNA species.^5^ Exosomes also possess a lipid bilayer membrane that grants the cargo it contains higher bioavailability and stability.^6^ Additionally, they also exhibit good biocompatibility and low immunogenicity, which increase their potential as therapeutic cargo carriers with an anti-inflammatory effect.^7^

 However, the clinical application of exosomes is beset by several challenges. Large quantities of exosomes are needed for clinical use, but translating research-grade cell culture processes into a clinical-grade protocol capable of being upscaled for mass production is time-consuming and expensive. Additionally, there is also the risk of contamination.^8^ Moreover, mammalian cell culture requires components (e.g., fetal bovine serum, FBS) that raise bioethical concerns and are generally prohibited during drug approval processes because of safety issues.^9,10^ Therefore, alternative exosome sources must be explored.

 Plants secrete extracellular vesicles with properties that are similar to those of their mammalian-derived counterparts.^11^ Plant sources are abundant, and their use raises minimal safety concerns. Therefore, plant-derived exosome-like nanoparticles (PELNs) can be obtained in higher quantities with lower costs.^12^ PELNs play a similar role to mammalian exosomes in terms of facilitating intercellular communication, and are known to also mediate inter-kingdom communication. In doing so, PELNs can exert therapeutic effects in humans that vary depending on the specific plant source,^13^ ranging from oxidative stress prevention to inflammatory gene expression modulation.^14,15^ Thus, PELNs may serve as a potential alternative therapeutic agent for overcoming the disadvantages associated with conventional anti-inflammatory drugs and mammalian-derived exosomes.

 The ginger rhizome is widely recognized for its health benefits and commonly used in herbal medicine. Its active components, such as 6-gingerol and 6-shogaol, exhibit anti-inflammatory activities, making them potential PELN sources for chronic inflammation treatment.^16^ Ginger-derived ELNs also ameliorate inflammation.^17,18^ However, different ginger varieties show different bioactive components and morphologies. Among the ginger varieties commonly cultivated in Indonesia, red ginger and emprit ginger are often used in traditional medicine and contain high levels of bioactive components. From these varieties, 6-gingerol and 6-shogaol and their potential as sources of anti-inflammatory PELNs have not yet been evaluated.^19^ Furthermore, ginger is readily available and relatively easy and inexpensive to obtain, making it a convenient and viable source for mass production.^20^ This study aims to characterize the ELNs derived from the rhizomes of Indonesian red ginger and emprit ginger varieties and determine their anti-inflammatory potentials by investigating their effects on the IL-6 secretion of RAW 264.7 macrophages.

## Materials and Methods

###  Materials and cell culture

 Murine macrophage RAW 264.7 cells were purchased from Elabscience (Houston, TX, USA) (CL-0190). The cells were cultured in high-glucose Dulbecco’s Modified Eagle Medium (DMEM) (Gibco [Waltham, MA, USA]; Sigma–Aldrich [St. Louis, MO, USA]) and supplemented with 10% (v/v) FBS (Gibco [Waltham, MA, USA]), as well as 1% (v/v) antibiotic–antimycotic solution (Ab–Am) (Gibco [Waltham, MA, USA]) at 37 ℃ under 5% CO_2_. The cells were then harvested mechanically with a cell scraper and subcultured upon reaching sub-confluent conditions.

###  Isolation of exosome-like nanoparticles from red ginger and emprit ginger

 RG-ELN and EG-ELN were isolated from the rhizomes of *Zingiber officinale *var. *rubrum *and *Zingiber officinale *var. *amarum*, respectively, following previously published polyethylene glycol-basedprotocols for ELN isolation,^21,22^ with slight modifications. Fresh rhizomes were purchased from a local market in Bandung, Indonesia. Roughly 300 g of rhizomes was washed, peeled, and homogenized to obtain ginger juice. The juice of both ginger variants were strained through a nylon mesh with 100 µm pore sizes to filter out the excess fibers before being sequentially centrifuged at 300 × *g* for 10 min, 2000 × *g*,6000 × *g* for 20 min, and 10 000 × *g* for 45 min at 4 ℃ to remove larger vesicles and particles. The supernatant of each variant was mixed thoroughly by rocking and inversion with a PEG6000 and NaCl solution to a final concentration of 10% (w/v) and 0.5 M, and then incubated overnight at 4 ℃. The mixture was then centrifuged at 8000 × *g *for 30 min at 4 ℃. After removing the supernatant, the tubes were allowed to drain for 5 min on a tissue paper to remove the excess liquid and PEG. Subsequently, the resulting pellets were resuspended in phosphate-buffered saline (PBS) and 25 mM trehalose, filtered through 0.45 and 0.22 µm Minisart® syringe filters (Sartorius [Göttingen, Germany]) to obtain RG-ELN and EG-ELN, respectively, and stored at −20 ℃ until further use.

###  Characterization of exosome-like nanoparticles

 The RG-ELN and EG-ELN morphologies were observed through transmission electron microscopy (TEM). The samples were loaded onto Formvar grids and left to absorb for 1 min before the excess was removed with filter paper. They were then negatively stained by adding UranyLess (Mauresac, France) staining solution onto the grids and leaving it to dry for 5 min. A Hitachi HT7700 instrument was used for visualization at 100 kV. Dynamic light scattering (DLS) using a Horiba SZ-100 instrument was done to determine the particle size distribution in the ELN samples. The samples were appropriately diluted, and 1.5 mL of the diluted samples was analyzed at 25 ℃. The total protein concentration acted as the basis for the ELN concentrations used in this study and was determined using the Pierce^TM^ BCA Protein Assay Kit (Thermo Fisher Scientific [Waltham, MA, USA]).

###  Assessment of RG-ELN and EG-ELN cytotoxicities

 The RG-ELN and EG-ELN cytotoxicities toward RAW 264.7 were determined by measuring cell viability using the MTT assay. RAW 264.7 cells were seeded in 96-well plates at a density of 1 × 10^4^ cells/well and incubated for 24 h. The cells were then incubated for another 24 h in DMEM + 5% (v/v) FBS with varying RG-ELN and EG-ELN concentrations ranging from 0 to 200 µg/mL. Absorbance after subsequent incubation with MTT reagent and addition of dimethyl sulfoxide (DMSO) were measured at 595 nm with an iMark^TM^ microplate reader (Bio-Rad).

###  Intracellular uptake of RG-ELN and EG-ELN

 The internalization of RG-ELN and EG-ELN into RAW 264.7 cells was assessed by labeling the ELNs with a PKH67 Green Fluorescent Cell Linker Kit (Merck) following the manufacturer’s protocol with modification. Subsequently, 125 µL of ELNs was incubated with 2 µL PKH67 and 125 µL Diluent C for 4 min at 25 ℃. To remove excess dye, the mixture was centrifuged at 20 000 × *g *at 4 ℃ for 60 min, and the supernatant was discarded. The pellets containing PKH-labeled ELNs were washed twice with ddH_2_O by centrifugation at the same speed for 15 min, and then resuspended in DMEM + 1% Ab–Am. RAW 264.7 cells were seeded on coverslips and placed in six-well plates 24 h before treatment with PKH-labeled RG-ELN and EG-ELN. Observation using an Olympus Fv1200 confocal laser scanning microscope was performed after a 2 h incubation period and subsequent fixation with 4% (w/v) paraformaldehyde and DAPI counterstaining (Invitrogen [Waltham, MA, USA]).

###  In vitro macrophage inflammatory assay

 The anti-inflammatory potentials of RG-ELN and EG-ELN were assessed through a macrophage inflammatory assay protocol based on existing literature^23,24^ with slight modifications. RAW 264.7 cells were first seeded at a 2 × 10^4^ cells/cm^2^ density in six- and 24-well plates for RNA isolation and conditioned medium collection, respectively. The cells were incubated in DMEM + 10% (v/v) FBS + 1% (v/v) Ab–Am for 24 h at 37 ℃ before the culture medium was replaced with DMEM + 5% (w/v) FBS containing RG-ELN (5, 10, or 50 µg/mL), EG-ELN (5, 10, or 50 µg/mL) or 1 µg/mL dexamethasone. After 24 h, the RAW 264.7 cells were stimulated by replacing the medium with DMEM + 5% (w/v) FBS containing 10 ng/mL LPS, followed by incubation for 6 h at 37 ℃. The cells were then either lysed for total RNA isolation and reverse transcription quantitative real-time polymerase chain (RT-qPCR) analysis, or its conditioned medium was collected for enzyme-linked immunosorbent assay (ELISA).

###  RT-qPCR analysis and ELISA for detection of IL-6 expression

 The IL-6 production was detected at the level of mRNA expression and protein secretion through RT-qPCR analysis and ELISA, respectively. For the RT-qPCR analysis, total RNA was extracted from RAW 264.7 cells using the Quick RNA Miniprep Plus Kit (Zymo Research [Irvine, CA, USA]) according to manufacturer protocols. The quantity and the purity of RNA were assessed using NanoDrop^TM^. RNA integrity was evaluated through agarose gel electrophoresis. Total RNA was stored at −80 °C until further use. cDNA was synthesized from 1 µg of total RNA, which, along with RT-qPCR, was done using the GoTaq® 2-Step RT-qPCR System (Promega [Madison, WI, USA]). RT-qPCR was performed using primers (Integrated DNA Technologies [Coralville, IA, USA]) targeting murine IL-6 (forward, 5′-ATCCAGTTGCCTTCTTGGGA-3′; reverse, 5′-GGTCTGTTGGGAGTGGTATCC-3′) and GAPDH (forward, 5′-TGTGTCCGTCGTGGATCTGA-3′; reverse, 5′-TTGCTGTTGAAGTCGCAGGAG-3′) on the CFX96 Touch Real-time PCR Detection System (Bio-Rad). The PCR efficiency was calculated from the fluorescence increase in the exponential phase with LinRegPCR.^25,26^ The relative RNA expression levels were calculated on Microsoft Excel using the Pfaffl method^27^ and expressed as the fold change in the gene expression relative to the stimulated macrophages with GAPDH as the endogenous control.^28,29^

 For cytokine secretion detection, the conditioned medium was collected and centrifuged at 1000 × *g* for 20 min at 4 ℃ to remove the cells and debris before analysis with Mouse IL-6 ELISA Kit (Elabscience [Houston, TX, USA]). The ELISA assay was performed following the manufacturer’s protocol. Absorbance was measured at 450 nm with a microplate reader (iMark^TM^, Bio-Rad).

###  Statistical analysis

 Data were reported as mean ± SD with n ≥ 3. The outliers were identified, and one-way analysis of variance with Tukey post-hoc was conducted using GraphPad Prism 9 Software (GraphPad Software, Inc.). The confidence intervals were set to 95% to determine the statistical significance.

## Results and Discussion

###  Isolation and characterization of RG-ELN and EG-ELN

**Figure 1:**
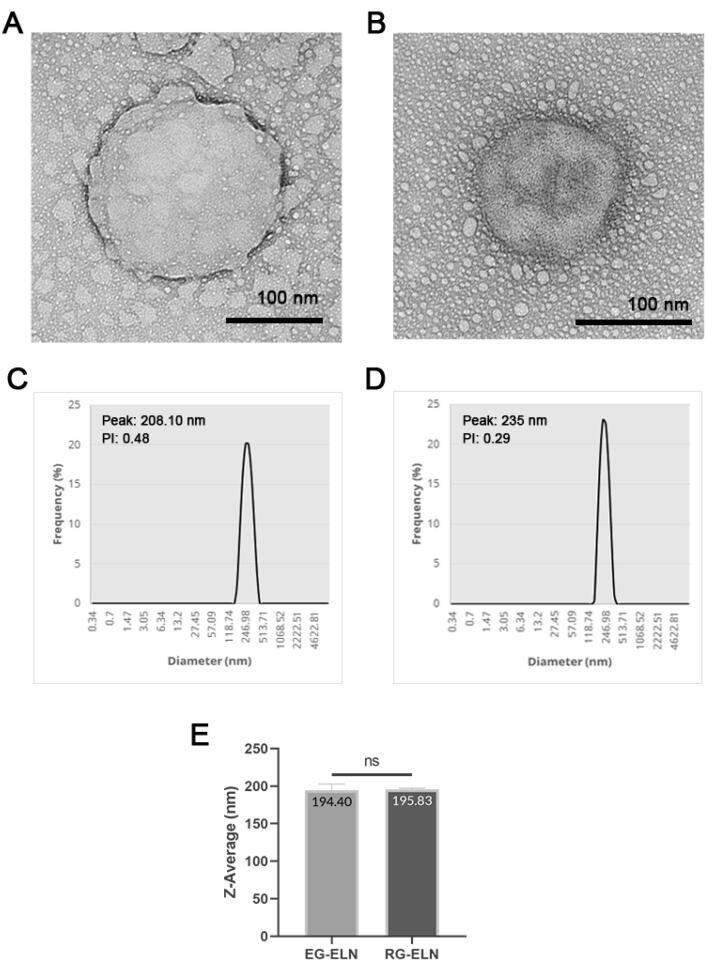
Morphology and size profile of RG-ELN and EG-ELN. TEM images of (A) EG-ELN and (B) RG-ELN negatively stained with Uranyless dye. Black bars indicate 100 nm. Size distributions of particles in (C) EG-ELN and (D) RG-ELN samples as well as (E) Z-average of EG-ELN and RG-ELN particles based on Dynamic Light Scattering (DLS). ns = not significant (*P* > 0.05)

 The samples of both PELNs exhibited cup-shaped/collapsed morphologies in line with previous exosome descriptions (Figure 1).^30^ The observed structures were stained darker at the edges, marking the accumulation of Uranyless dye on the background caused by the repulsion between the similarly negatively charged dye and the phosphate groups of the phospholipid bilayer membrane of the exosome membrane.^31^ This indicated the presence of particles with intact membranes.^32^ The cup-shaped or collapsed morphologies of extracellular vesicles (EVs) when visualized with TEM might be an artifact of the sample preparation resulting in EVs not being in their native spherical configuration.^33^ Previous studies have also reported ginger ELNs with a similar morphology.^17,34,35^

 DLS was conducted on the PELN samples to ascertain the particle size distribution within the samples. The particles contained within the representative samples of both RG-ELN and EG-ELN were polydisperse with PI values > 0.1^36^ and range within 150–400 nm in size with average sizes at 195.83 ± 1.36 and 194.40 ± 8.40 nm, respectively (Figure 1). The size profile of RG-ELN and EG-ELN fell within the range of previously reported size of PELNs (50–500 nm). The independent *t*-tests did not indicate significant differences in the average size of the particles between the RG-ELN and EG-ELN samples (*P* > 0.05).^37^ The total protein concentrations of the RG-ELN and EG-ELN samples as per the BCA assay were 3326.1 ± 63.8 and 1252.5 ± 37.4 µg/mL, respectively. The ginger ELNs isolated using the polymer precipitation-based methods of Kalarikkal et al^22^ had larger-sized particles on average (~300 nm) and an overall wider size distribution, with maximum sizes reaching 900 nm. The notable difference in the average size and range of particles was possibly caused by the use of 0.22 µm syringe filters as a part of the ELN isolation process in this study. Both the RG-ELN and EG-ELN samples were polydisperse in nature, which is in line with the heterogeneity of exosomes in literature. PELNs have also been reported with PI values of 0.2–0.4.^12,34^

###  Effect of PELNs on RAW 264.7 cell viability

**Figure 2:**
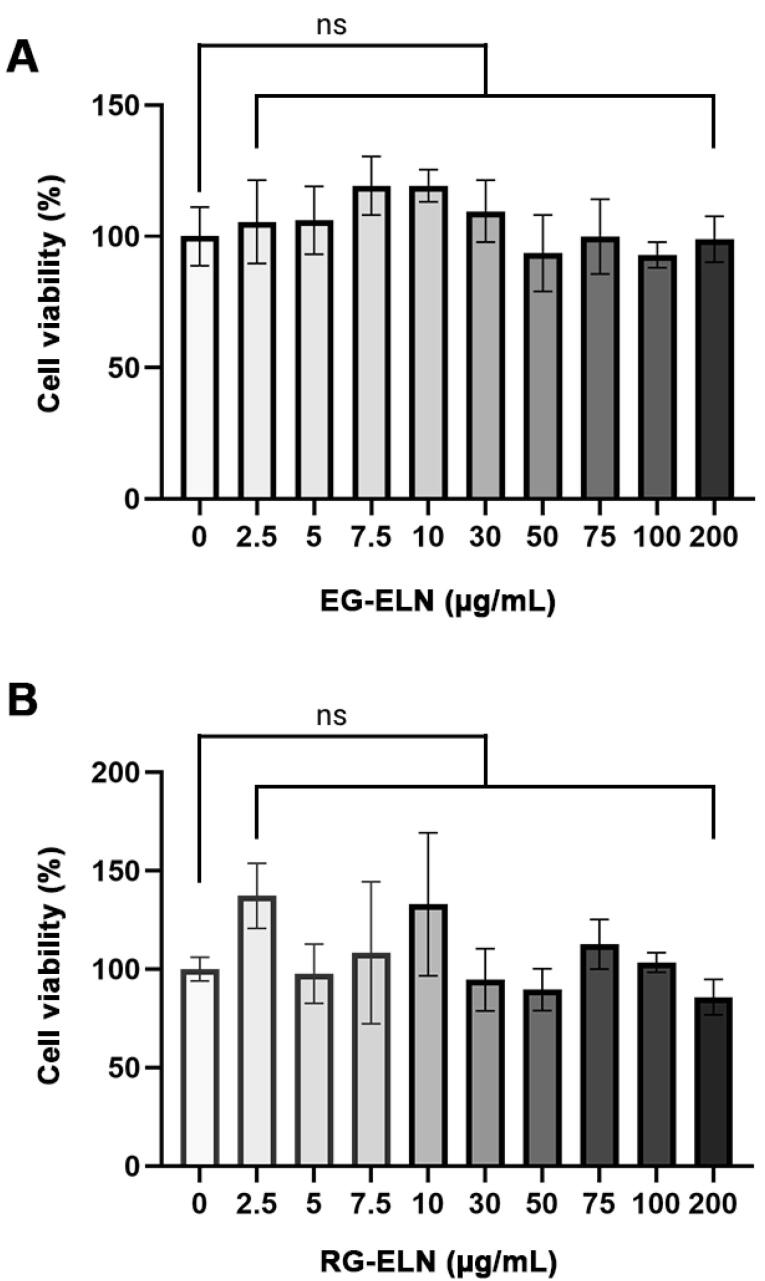
Cytotoxicity of PELNs. Effect of different concentrations of (A) EG-ELN and (B) RG-ELN towards percentage of live RAW 264.7 cells relative to control group (0 μg/mL). Viability was calculated based on MTT assay. Data is displayed as mean ± SD (n = 5). No significant difference in cell viability between 0 μg/mL group and 2.5, 5, 7.5, 10, 30, 50, 75, 100, 200 μg/mL groups for both EG-ELN and RG-ELN. ns = not significant (*P* > 0.05)

 In this study, RAW 264.7 was used to assess the anti-inflammatory potential of PELNs in vitro. As such, the cytotoxicities of RG-ELN and EG-ELN toward the cells must first be evaluated. PELNs are natural nanoparticles sourced from edible plants, such as ginger; thus, they exhibit low toxicity.^9^ To verify this, the effects of RG-ELN and EG-ELN at various concentrations on the viability of RAW 264.7 macrophages after a 24 h incubation period were determined through the MTT assay. Compared to the untreated controls (0 µg/mL), treatments of either PELN at up to 200 µg/mL concentrations did not significantly affect RAW 264.7 cell viability (*P* > 0.05) (Figure 2). The results indicate that RG-ELN and EG-ELN neither exhibited toxicity nor induced the proliferation of RAW 264.7 macrophages at the tested concentrations.

 The cytotoxicity of PELNs vary depending on the specific plant source, but is generally said to have a lower toxicity when compared to mammalian-derived exosomes.^38^ Ginger is a plant widely consumed as a spice or as a form of traditional medicine, which may contribute to the low toxicity of ginger ELNs.^19,20,39^ The results obtained in this study were in alignment with those of previous reports on ginger ELNs by Zhang et al^17^ which showed no effect on RAW 264.7 viability at up to 100 µg/mL concentrations. The same study also reported no adverse side effects or toxicity upon oral administration of ginger ELNs. Furthermore, the low toxicity of PELNs makes it advantageous as a therapeutic agent when compared to synthetic alternatives which are associated with higher toxicity.^13^

###  Intracellular uptake of PELNs by RAW 264.7 cells

**Figure 3:**
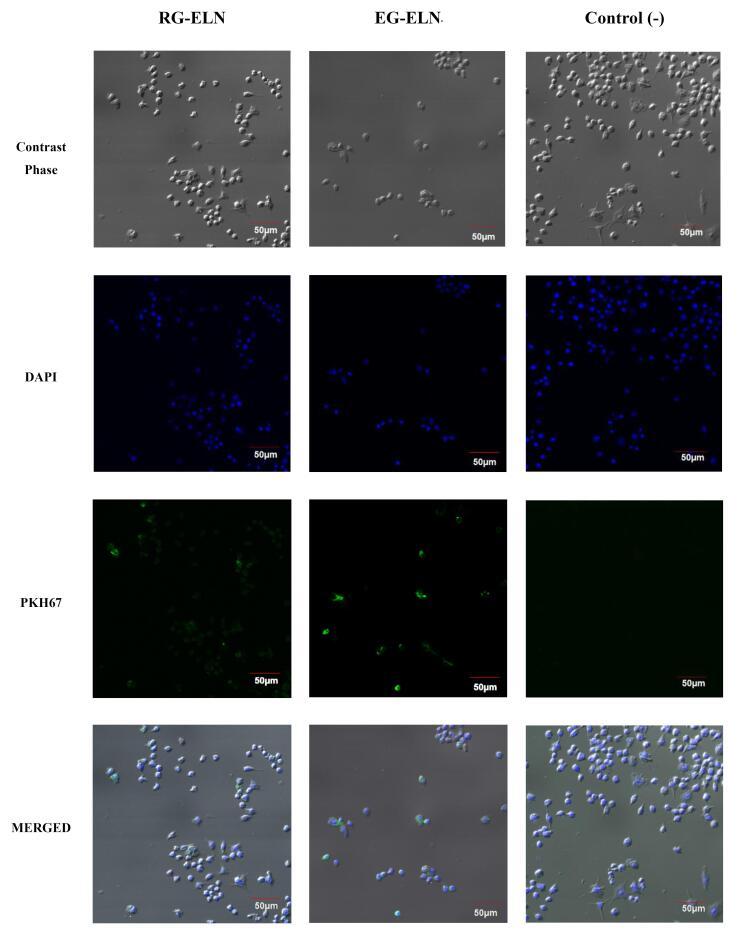
Intracellular uptake of RG-ELN and EG-ELN. Confocal images depicting uptake of RG-ELN and EG-ELN 2 h following treatment. PELN was labelled with green fluorescent dye PKH67, nuclei of RAW 264.6 cells were stained blue with DAPI, and cell morphology can be observed through phase contrast. Results compared with a mock-treated negative control group

 The intracellular uptake of PELNs is an important aspect to consider when assessing PELNs as a therapeutic agent. PELNs exert therapeutic effects upon internalization and subsequent cargo release.^40^ Thus, the internalization of particles in the representative samples of RG-ELN and EG-ELN was assessed by labeling PELNs with a PKH67 green fluorescent dye. Figure 3 shows the presence of PKH67-labeled PELNs within the cytosolic region of the macrophage cells. Within 2 h, the particles from the RG-ELN and EG-ELN samples have begun to internalize into the RAW 264.7 cells.

 Generally, exosomal uptake is known to be facilitated by several mechanisms, including plasma membrane fusion, clathrin-dependent endocytosis pathways, and clathrin-independent pathways (e.g., macropinocytosis and lipid raft-mediated endocytosis).^41^ In addition, phagocytic cells like RAW 264.7 may also be capable of internalizing exosomes through phagocytosis. Previous studies reported on the uptake of ginger-derived ELNs by intestinal Caco-2 cells through caveolin -mediated endocytosis and micropinocytosis and by hepatocytes through microtubule-dependent active transport.^18,42^ In this study, RG-ELN and EG-ELN were internalized by RAW 264.7 macrophages within 2 h following treatment. Zhang et al^17^ reported the uptake of ginger ELNs by the same cells when observed 4 h after incubation, meanwhile Chen et al^43^ observed the internalization of ginger ELNs by bone marrow-derived macrophages at 16 h following treatment. A previous study by Kalarikkal et al^22^ demonstrated the uptake of ginger ELNs as early as 10 min post-addition and saturated at 8 h. This study was limited to observation after a set amount of incubation period; thus, it might be possible for RG-ELN and EG-ELN to be internalized within a shorter amount of time, as well.

###  Effect of RG-ELN and EG-ELN on IL-6 secretion of LPS-stimulated RAW 264.7 macrophages

 After the presence of ELNs within the RG-ELN and EG-ELN samples was confirmed through the previously discussed characterization steps, the anti-inflammatory potential of PELNs was assessed *in vitro *through their ability to inhibit the cytokine production in LPS-activated macrophages.^44^ In this study, the anti-inflammatory potentials of RG-ELN and EG-ELN were investigated *in vitro *through a macrophage inflammatory assay. Macrophages are versatile cells capable of switching to a pro-inflammatory phenotype upon exposure to signals associated with the presence of pathogens, such as LPS. Pro-inflammatory macrophages produce pro-inflammatory cytokines involved in initiating and sustaining the inflammatory response. Macrophages can also shift toward a pro-resolving phenotype marked by producing anti-inflammatory cytokines.^44^ In this way, macrophages play a crucial role in the initiation, progression, and resolution of inflammation.^45^ Thus, the ability of a tested sample to prevent macrophage polarization into a pro-inflammatory phenotype and alter the production of pro-inflammatory cytokines indicates its potential as a therapeutic agent for inflammatory disease treatment.^23^ IL-6 is among the pro-inflammatory cytokines secreted by activated macrophages and plays a key role in chronic inflammation. Inhibiting IL-6 signaling is considered an effective strategy for the prevention and management of inflammatory diseases like arthritis and colitis.^46^

**Figure 4:**
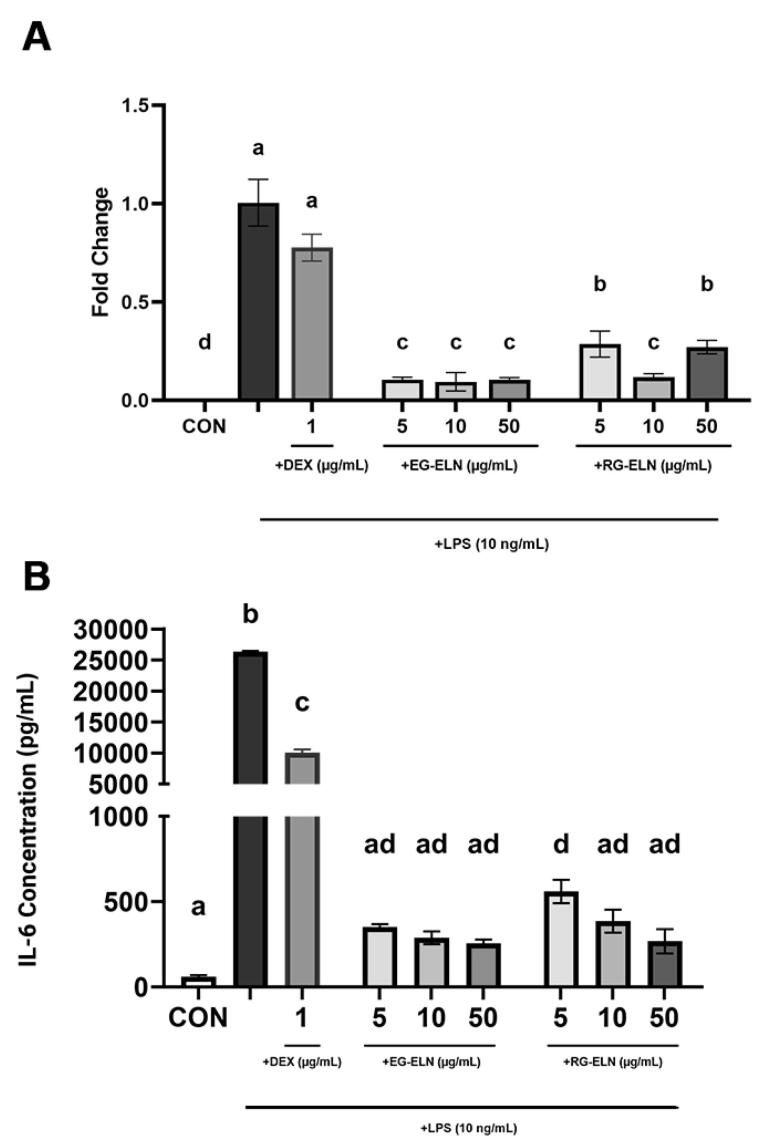
Effect of RG-ELN and EG-ELN on IL-6 production by LPS-activated RAW 264.7. Cells were treated with different concentrations of EG-ELN and RG-ELN followed by LPS activation. IL-6 production was analyzed at the level of (A) mRNA and (B) protein using qRT-PCR and ELISA respectively. qRT-PCR data was normalized against GAPDH with LPS only group (positive control) as the calibrator. Data is displayed as mean ± SD (n = 3). Bars not sharing any letter are significantly different (*P* < 0.05; Tukey). CON = negative control group

 Both RG-ELN and EG-ELN treatment groups exhibited significantly lower IL-6 production at the mRNA and protein levels when compared to activated controls (Figure 4). The IL-6 protein levels in the RG-ELN and EG-ELN treatment groups were significantly lower than those in the dexamethasone group. In other words, the anti-inflammatory potential of RG-ELN and EG-ELN at the lowest tested concentration (5 µg/mL) is greater or equal to 1 µg/mL of the commercial anti-inflammatory drug dexamethasone.

 The IL-6 concentrations in several RG-ELN (10 and 50 µg/mL) and all EG-ELN groups also did not significantly differ to the IL-6 concentrations of the negative control group. The results indicate that pre-treatment with either PELN at the tested concentrations suppressed IL-6 production in the LPS-activated macrophages to the extent that they did not differ from the unstimulated controls. This demonstrated the ability of both PELNs in preventing the acquisition of the pro-inflammatory macrophage phenotype. However, a clear trend between the degree of IL-6 suppression and the PELN dose could not be observed. The cytokine concentrations between the dose groups of RG-ELN or EG-ELN did not differ significantly. In addition, there also did not seem to be a significant difference in IL-6 secretions when comparing the RG-ELN and EG-ELN groups at the same concentration.

 The mechanism by which RG-ELN and EG-ELN can suppress IL-6 remains to be elucidated. However, in the LPS-stimulated macrophages, the expression of pro-inflammatory cytokines, such as IL-6, is mediated by the NF–κB pathway.^44^ Therefore, the anti-inflammatory mechanisms of RG-ELN and EG-ELN might involve the inhibition of components within the NF–κB signaling cascade. 6-Shogaol and 6-gingerol are major bioactive compounds found in ginger rhizomes with known anti-inflammatory activities.^16^ Previous research demonstrated that 6-shogaol inhibits IκB phosphorylation, preventing the degradation of IκB and maintaining the inhibition of the NF–κB complex. 6-Shogaol can also interfere with NF-κB signaling through Nrf2 activation. Overall, the actions of 6-shogaol prevent the translocation of the NF–κB complex to the nucleus and the subsequent transcription of pro-inflammatory genes, including IL-6.^47^ Zhang et al^17^ previously reported ginger ELNs to contain high concentrations of 6-shogaol and 6-gingerol, and given that these compounds are found in higher amounts in red ginger and emprit ginger varieties compared to the common ginger variety (*Z. officinale *var. *officinale*),^19^ it is possible that RG-ELN and EG-ELN also enclose these compounds as a part of their cargo. The bioactive compounds within ELNs are protected from degradation,^6^ which is an advantage ELNs have over plant extracts as extract usage is limited by the stability of its contents, which might face degradation before reaching the intended cells or tissues.^48,49^ The anti-inflammatory potential of RG-ELN and EG-ELN may also be mediated by other cargos, such as miRNAs. A study by Yin et al^18^ uncovered that the majority of miRNAs enriched in ginger ELNs were involved in several pathways, including inflammation regulation.

 Several studies regarding the anti-inflammatory potential of ELNs from various plants have been conducted. ELNs from plants such as apples, grapes, cabbages, grapefruit, broccoli, garlic, lemon, and onions have demonstrated anti-inflammatory potential.^13,24,48^ Studies specifically investigating the anti-inflammatory activities of ginger-derived PELNs was also previously conducted. Yin et al^18^ reported a reduction of IL-6 production in LPS-induced intestinal cells. Oral administration of ginger ELN (0.3 mg/mouse) by Zhang et al^17^ also managed to decrease pro-inflammatory cytokine production in a mouse model of colitis. However, this present study is the first to examine the anti-inflammatory potential of specific varieties of gingers, namely, the Indonesian red ginger and the emprit ginger as an ELN source. This study demonstrated that PELN derived from these varieties also possess a significant anti-inflammatory potential. For the purpose of mass production, the uniformity of source material is important in ensuring the consistency of the resulting products. Therefore, the results presented in this work highlighted the opportunity for these two cultivated varieties as PELN sources and might be considered in determining the specific varieties most suitable for the production of therapeutic agents for inflammatory disease treatment.

 This study is also the first, to our knowledge, to assess the anti-inflammatory potential of ginger ELNs isolated using a polymer precipitation-based method. PEG is a water excluding polymer, which alters EV solubility, allowing the collection of EVs without using an ultracentrifuge.^50^ Polymer precipitation using PEG is relatively cost-effective and simpler because it does not require specialized equipment, thus making the method easier to upscale for producing large quantities of PELN.^51^ A previous study by Kalarikkal et al^22^ reported similar characteristics between PELN isolated through ultracentrifugation and PEG precipitation. The results of this study also further support the utility of this isolation method for producing PELNs by demonstrating the anti-inflammatory capacity of ginger ELNs isolated through this method.

 However, it bears mentioning that this study is not without its own limitations. This study was intended as an initial study for examining the potential of EG-ELN and RG-ELN as anti-inflammatory agents; thus, certain procedures were conducted only to the extent necessary to provide preliminary information. The assessment of the EG-ELN and RG-ELN cytotoxicities only encompassed a 24 h period. Although sufficient to provide initial evidence of the safety of the ELNs, it will be beneficial to investigate the effects of the long-term exposure of ELNs with longer incubation periods in future research. The contents and cargo of EG-ELN and RG-ELN have also yet to be investigated to further confirm the mechanism by which the ELNs exert their anti-inflammatory effects.

## Conclusion

 In this study, we successfully isolated ELNs from the rhizomes of the Indonesian red ginger and emprit ginger varieties (*Zingiber officinale *var. *rubrum *and *Zingiber officinale *var. *amarum* respectively) through polymer precipitation using PEG6000 and assessed their anti-inflammatory potential. The average size distributions of RG-ELNs and EG-ELNs were 195.83 ± 1.35 and 194.40 ± 8.40 nm, respectively. Both ELNs exhibited cup-shaped morphologies, were internalized by RAW 264.7 cells within 2 h, and did not significantly alter cell viability up to concentrations of 200 µg/mL. RG-ELN and EG-ELN significantly suppressed IL-6 production in activated RAW 264.7 macrophages. Overall, the study results demonstrate the potential use of Indonesian red ginger and emprit ginger varieties as a plant source and PEG precipitation as a method of ELN isolation that may be considered for the future production of PELNs as a form of treatment for inflammatory diseases.

## Competing Interests

 The authors have no conflict of interest.

## Ethical Approval

 Not applicable.
